# Supplementary material for: Resting-State Isolated Effective Connectivity of the Cingulate Cortex as a Neurophysiological Biomarker in Patients with Severe Treatment-Resistant Schizophrenia
Source: J Pers Med. 2020 Aug 14;10(3):89. doi: 10.3390/jpm10030089 (PMC7564631; doi:10.3390/jpm10030089)
Supplement: Supplementary file 1 [file jpm-10-00089-s001.pdf]

Supplementary

Table S1. MNI coordinates.

Seed ROIs		MNI coordinates		
		x	y	z
posterior cingulate cortex (PCC)	L	-10	-45	24
	R	9	-45	24
anterior cingulate cortex (ACC)	L	-5	1	32
	R	5	5	31

Montreal Neurological Institute (MNI) coordinates in the present study were based on previous articles [Park 2009, Moreines 2017, Lacadie 2008].

Table S2. Summary of significant results of 4-way ANOVA.

Analysis	Results
4-way ANOVA	
Main effects	
frequency	$F_{5, 225} = 34.39$, $p < 0.001$
Interactions	
frequency-by-directionality	$F_{5, 225} = 3.113$, $p = 0.010$
laterality-by-directionality-by-group	$F_{1, 45} = 6.619$, $p = 0.013$
frequency-by-laterality-by-directionality-by-group	$F_{5, 225} = 3.497$, $p = 0.005$
3-way ANOVA (Left)	
Main effects	
frequency	$F_{5, 225} = 22.20$, $p < 0.001$
Interactions	
directionality-by-group	$F_{1, 45} = 4.413$, $p = 0.041$
frequency-by-directionality	$F_{5, 225} = 3.369$, $p = 0.006$
2-way ANOVA (Left, delta frequency)	
Interactions	
frequency-by-laterality	$F_{5, 225} = 3.391$, $p = 0.006$
Post-hoc independent t-tests for the ratio of directionality between the PCC and ACC	
Delta band in the left side	$t_{45} = 7.659$, $p = 0.008$
Theta band in the left side	$t_{45} = 8.066$, $p = 0.007$
